# Artificial intelligence in genomics: transforming the diagnosis of hereditary spastic paraplegia

**DOI:** 10.1097/MS9.0000000000003882

**Published:** 2025-08-15

**Authors:** Hafiza Aimal Nizami, Alishba Rafiq, Maliha Khalid, Aminath Waafira

**Affiliations:** a Department of Medicine, Dow University of Health Science, Karachi, Pakistan; b Department of Medicine, Jinnah Sindh Medical University, Karachi, Pakistan; c School of Medicine, The Maldives National University, Malé, Maldives

**Keywords:** artificial intelligence in genomics, hereditary spastic paraplegia (HSP), next-generation sequencing (NGS), variant pathogenicity prediction

## Abstract

Hereditary spastic paraplegia (HSP) represents a genetically and clinically heterogeneous group of neurodegenerative disorders characterized by progressive spasticity and weakness of the lower limbs. Accurate diagnosis remains challenging due to the expanding spectrum of causative genes, overlapping features with hereditary cerebellar ataxias, and the presence of clinical mimics. Recent advances in artificial intelligence (AI)-driven genomics have begun to transform variant detection, interpretation, and pathogenicity classification. Accelerated next-generation sequencing platforms such as NVIDIA Parabricks and Illumina DRAGEN markedly shorten analysis times while maintaining clinical-grade accuracy. Emerging tools such as Dynamicasome, which integrates molecular dynamics simulations with deep learning, address long-standing limitations by capturing structural disruptions beyond sequence conservation. Complementary approaches like SpliceAI and Exomiser expand the diagnostic window through improved splicing prediction and phenotype-driven variant prioritization. These innovations are particularly relevant in high-consanguinity populations such as Pakistan, where recessive variants are prevalent and diagnostic delays are common. Wider adoption of free AI-based platforms, alongside bioinformatics training and institutional collaboration, has the potential to accelerate early molecular diagnosis, enable timely genetic counseling, and ultimately reshape the clinical management of HSP and other rare neurogenetic disorders.

*Dear Editor,*

Hereditary spastic paraplegia (HSP) is a group of rare genetic neurological disorders represented by progressive spasticity and weakness of the lower limbs. Globally, its prevalence is approximately 3.6 per 100 000, and more than 100 genetic subtypes of HSP have been reported so far^[1]^. The diagnosis of HSP remains challenging, due to a rapidly expanding catalogue of implicated genes, phenotypic overlap with disorders such as hereditary cerebellar ataxia, and the presence of clinical mimics. Furthermore, a single gene defect can manifest through diverse inheritance patterns and highly variable phenotypes extending to non-neurological complications. These challenges with scarcity of specialized expertise in rare diseases frequently lead to misdiagnosis or delayed recognition. Variability in age of onset, rate of progression, mode of inheritance (as all modes of inheritance can be observed in HSP, i.e., X-linked recessive, autosomal recessive, autosomal dominant, and mitochondrial), symptom severity, and clinical course vary widely across different HSP subtypes^[1]^, further complicating clinical recognition.

The landscape of genomic variant analysis has been revolutionized by artificial intelligence (AI)-driven tools – from detection to interpretation – with increasing speed and accuracy. 2025-AI-driven platforms have transformed genomic variant calling tools with unmatched speed and clinical-grade accuracy, like accelerated Next Generation Sequencing analysis pipelines, specifically NVIDIA parabricks (leveraging GPU-optimized HaplotypeCaller, DeepVariant Calling) and Illumina DRAGEN (powered by FPGA-based Germline Small Variant Caller)^[2]^. Parabricks on H100 GPUs is 75–126 times faster than Central Processing Unit (CPU) pipelines with >99.8% Single Nucleotide Variant (SNV) and >99.1% indel accuracy, while DRAGEN achieves 49–78 times speedups^[2]^. Traditional CPU-based pipelines take days per sample, but these AI tools overcome this by using machine learning for error suppression with algorithmic innovation like genome partitioning, enabling workflow up to 329 times faster in hours^[2]^.

In 2025, Dynamicasome, a breakthrough approach integrating molecular dynamics simulations (MDS) with AI-driven modelling, now enables precise, accurate pathogenicity classification of genetic variants^[3]^. By simulating atomic-level structural disruptions caused by mutations, capturing dynamic changes invisible to sequence-based tools and training Dynamic Neural Networks (DNNs) on these biophysical features, we have achieved unprecedented accuracy in distinguishing pathogenic from benign variants^[3]^. Biologically validated in vivo, this MDS–AI model resolves Variants of Uncertain Significance and overcomes critical limitations of conventional predictors (REVEL, AlphaMissense), which rely primarily on static sequence conservation and lack sensitivity to subtle structural and functional disruptions. This protein–motion–centric paradigm directly accelerates accurate diagnosis of rare diseases.

Moreover, SpliceAI and Exomiser are some other AI tools that are transforming other crucial steps of genomic analysis. SpliceAI is a deep learning model that allows for the accurate identification of noncoding genomic variations that may result in cryptic splicing^[4]^. It widens the range of diagnosis for genetically diverse conditions such as HSP. Exomiser integrates both variant and phenotype-associated data to produce a single combined score or likelihood for potential variations^[5]^. It cross-references the patient’s phenotypes with known phenotypic patterns from human diseases and model organisms related to candidate genes. Exomiser accelerates the diagnosis process by quickly identifying the most likely causative gene out of more than 100 potential candidates.

These tools have a revolutionary effect on the genetic complexity of HSP. In consanguinity-prone populations like Pakistan, these AI-driven pipelines can improve recognition of recessive variants and reduce diagnostic delays. The availability of free AI tools like DEEPVARIANT, CADD, REVEL, and SpliceAI makes the integration of AI more affordable in middle-income countries like Pakistan. Early molecular detection provides genetic counseling and directs family planning. We advocate integrating AI-based genomics in clinical neurology, along with the training of physicians and laboratory personnel in bioinformatics. The skill gap might be reduced with the help of institutional partnerships and open-access training. We must seize the opportunity provided by the data-driven genomics to simplify complex neurological diseases like HSP. Our work is in line with the TITAN Guidelines on the need for transparency in AI use in healthcare^[6]^.

## Data Availability

No data were generated for this manuscript.

## Research registration unique identifying number (UIN)

(1) Name of the registry: N/A. (2) Unique Identifying number or registration ID: N/A. (3) Hyperlink to your specific registration (must be publicly accessible and will be checked): N/A.
